# A high-absorbance water-soluble photoinitiator nanoparticle for hydrogel 3D printing: synthesis, characterization and in vitro cytotoxicity study

**DOI:** 10.1038/s41598-023-35865-3

**Published:** 2023-05-26

**Authors:** Hanieh Sadat Ghazali, Esfandyar Askari, Amir Seyfoori, Seyed Morteza Naghib

**Affiliations:** 1 Nanotechnology Department, School of Advanced Technologies, Iran University of Science and Technology, Tehran, Iran; 2 Biomaterials and Tissue Engineering Department, Breast Cancer Research Center, Motamed Cancer Institute, ACECR, Tehran, Iran

**Keywords:** Biomaterials, Biomaterials, Tissue engineering

## Abstract

Light-based hydrogel crosslinking is a new approach in rapid and high-resolution 3D printing; however, using this method in tissue engineering is challenging due to the toxicity of photoinitiators, their solvents, and their low efficiency. Herein, a novel, water-soluble photoinitiator with high efficiency in light-based 3D printing is introduced. Low-cost photoinitator, 2,4,6-trimethylbenzoylphenyl phosphinate, is converted into nanoparticles via a microemulsion method and dispersed in water. Cell toxicity assays were performed to prove that these nanoparticles are non-toxic and can be used in biomedical applications. Finally, the nanoparticles were utilized in the high-accuracy 3D printing of hydrogels. The results of this study indicate that these particles are potent to be used in bioprinting.

## Introduction

Utilizing light in biofabrication has gained interest owing to the accuracy and speed this method brings^1,2^. Recent developments in hydrogel 3D printing and soft tissue engineering are mainly focused on light-based crosslinking approaches. Light-based 3D printing enables layer-by-layer fabrication of pre-designed geometries and interconnected structures for tissue regeneration by using photo crosslinkable materials^3,4^. Digital light processing (DLP) is a technique categorized in light-based 3D printing. In this technique a laser beam of light source (LED or UV projector) is used to cure a polymer in a top-down process. Some DLP devices are equipped with a digital mirror device (DMD) to control the on and off state of the curing laser. In this technique, a whole layer of the pattern is cured instantly which makes this technique fast and accurate^5,6^. Light-based 3D printing of hydrogels is a specific asset in tissue engineering as they are ideal for cell encapsulation, and cell expansion, and do not require an intolerable pH condition or high temperature^7,8^. Also, in the photopolymerization of hydrogels, the properties of final structures, either mechanical or chemical, can be altered by crosslinking factors such as light intensity or crosslinker concentration^9,10^. The photoinitiator (PI) is the main and critical component that should be considered to initiate the photopolymerization of a hydrogel, oligomer, or monomer^11^.

Applying light-based technologies in the field of tissue engineering and biofabrication requires some vital considerations. The critical wavelength of light that can activate the PI, stability in aqueous solution, and photoactivity are crucial criteria that ignoring them in the preparation of ink cause major problems including cytotoxicity, inefficient cell adhesion, and genotoxicity of cells. Water solubility is the main factor, as organic solvents cannot provide a suitable environment for cells and other biomolecules; heating or stirring the precursor may also cause damage to them. Moreover, efficiency is another factor that should be considered in printing^12^. A slow photopolymerization rate can be harmful to other components as it needs a longer exposure time. Also, different PIs undergo different photopolymerization methods, which affects the final structure's homogeneity^13^. There are limited numbers of PIs that are water-soluble and biocompatible^14–17^. The most common PI in tissue engineering applications is Irgacure 2959 (I2959). I2959 is less toxic than other commercially available PIs; however, this PI's limited water solubility and efficiency are significant drawbacks ^12,18,19^. Recently, 2,4,6-trimethyl benzoyl diphenyl phosphine oxide (TPO) has been used for photopolymerization. This PI can absorb and work under UV and visible irradiation, it is a highly efficient PI, provides accurate and high-speed light-based 3D printing, yet it is not water-soluble^3,20,21^.

This study introduces high-efficient, water-soluble, and non-toxic UV light photoinitiator nanoparticles obtained from pristine TPO for use in light-based 3D bioprinting. TPO nanoparticles (TPO NPs) were synthesized by using the emulsion-based technique in which SDS as a surfactant inhibits the growth of TPO nanoparticles. X-ray diffracyion (XRD), UV–Visible spectroscopy (UV–Vis), and field emission scanning electron microscopy (FE-SEM) were used to study the physiochemical properties of synthesized NPs, and they confirmed the successful formation of TPO NPs. According to our literature review, there is no reported study about the cytotoxicity of TPO as the photoinitiator. We used MTT and live–dead assays to study the effect of TPO NP’s concentration on the viability of HUVEC and MCF-7 cells in 2D monolayer and 3D cell spheroid conditions. Cell apoptosis was also studied by using flow cytometry to better understand how NPs interact with the cells. Finally, as proof of concept, TPO NPs were used to 3D print photo crosslinkable bioresin including PEGDA, GelMA, and food dye. For this purpose, a DLP was used which enables us to print different geometries with high accuracy.

## Materials and methods

### Materials

2,4,6-trimethyl benzoyl diphenyl phosphine oxide (TPO), Gelatin, Methacrylic anhydride (MAA), Poly(ethylene glycol) diacrylate (PEGDA, Mn 700), *N*-butyl acetate (nBuAc), 2-propanol (IPA), sodium dodecyl sulfate (SDS), polyvinylpyrrolidone (PVP), Polydimethylsiloxane (PDMS), sodium carbonated, sodium bicarbonate, sodium hydroxide, hydrochloric acid, DAPI and Live/Dead viability/cytotoxicity kit including PI and annexin were purchased from Sigma Aldrich. Dulbecco's Modified Eagle's Medium (DMEM), fetal bovine serum (FBS), pen-strep, and trypsin were purchased from Gibco. Collagenase was obtained from Merck. All of the reagents were used without any purification.

### Synthesis of TPO nanoparticles

**Figure 1:**
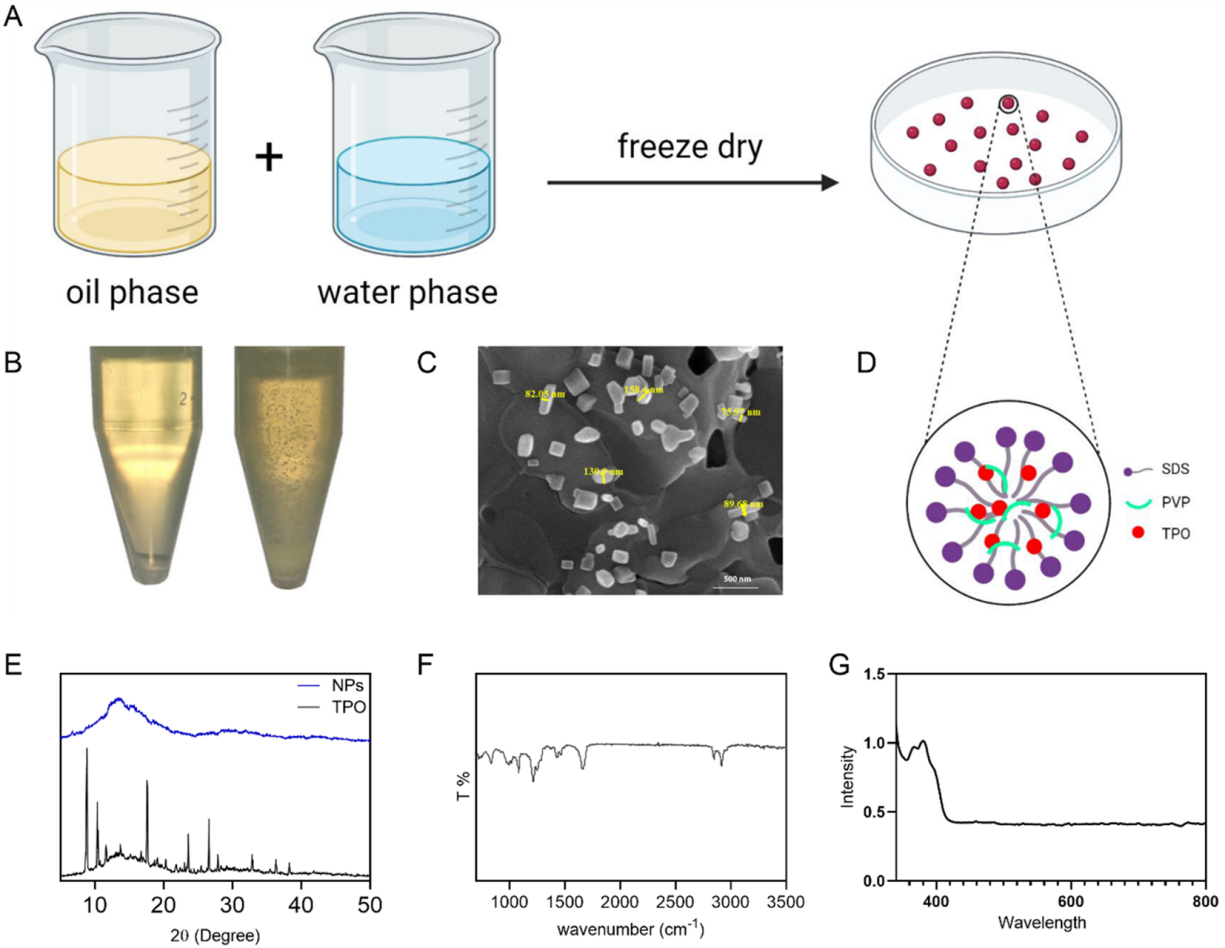
Characterization of TPO NPs: (**A,B**) TPO NPs dissolved in water (left) and TPO bulk suspended in water (right), (**C–E**) XRD patterns from TPO bulk and TPO NPs, FE-SEM image of TPO NPs, (**F**) FT-IR spectrum of TPO NPs, structure of resultant TPO NPs after drying, and (**G**) UV–Vis spectrum of TPO NPs dissolved in water, a schematic of synthesis method.

Purchased TPO was converted to highly water-soluble nanoparticles utilizing the micro-emulsion method^22^. In this method (schemed in Fig. 1A), evaporation of the volatile solvent in Water/Oil micro-emulsion, nBuAc, allows the formation of nanoparticles. Briefly, W/O micro-emulsion was prepared by mixing 22.3%(w/w) nBuAc as a volatile solvent and 7.5%(w/w) SDS as a surfactant at room temperature and continuously stirred with a magnetic stirrer at 250 rpm. After dissolving, 21%(w/w) IPA as co-solvent and 7.5%(w/w) PVP as crystallization inhibitor were added to the solution under the same condition, respectively. Then, 1.7%(w/w) TPO is added to the solution. The solution was mixed with 40%(w/w) TDW until the micro-emulsion became clear. The nanoparticle powders were obtained by lyophilization of the solution, which was performed using an FD-10 freeze-dryer (Pishtaz Engineering company, Iran) at −65 °C for 24 h. Obtained powders can be stored at −20 °C.

### GelMA synthesis

Shirahama et al. have reported a novel-effective route for GelMA synthesis^23^. In short, 10% w/v type A Gelatin (175 bloom) was dissolved in 0.1 M Carbonate-Bicarbonate (CB) buffer, which comprised 3.18 g sodium carbonate and 5.86 g sodium bicarbonate in 1L distilled water. After adjusting pH with 5 M sodium hydroxide or 6 M hydrochloric acid on 9, MAA (0.1% w/w Gelma) was added to the solution under a magnetical stirrer at 400 rpm. After 3 h, the reaction was stopped by adjusting the pH to 7.4. The final solution was filtered, dialyzed (24 h) using a 12–14 kD dialyze tube, and lyophilized for 24 h. the samples can be stored at −20 °C until further use.

### Preparation of photocurable resin

For using nanoparticles in 3D printing, 1–2% w/v of obtained powders was added to the aqueous solution. We also investigated the effect of GelMA concentration ranging from 10, 15, and 20% with all samples containing PEGDA (10% w/w GelMA as co-crosslinker). Food dye was also used as a photo absorber.

### 3D printing parameters, optimization of curing time

A mold was printed using a custom-made DLP 3D printer to optimize the curing time of hydrogels. The printed mold was spattered with gold and filled with PDMS. After the gelation of PDMS, a 30 µm well was created. The prepared batches were tested under UV light (365 nm wavelength) using microwells in order to report the curing time.

### Characterization

#### FE-SEM, XRD, UV–vis, FT-IR, H-NMR

The size distribution of synthesized nanoparticles was measured by field emission scanning electron microscopy (TE-SCAN VEGA instrument). Nanoparticle powder XRD measurement was performed using INEL EQuinox 3000 instruments with 40 kV, 30 mA, and 1.541874 Å wavelength. Ultraviolet–visible (UV–vis) absorption spectra of GelMA and TPO nanoparticles were measured by Biowave II spectrometer instruments. Also, it was performed on 1 mg/ml of TPO nanoparticles aqueous solution, and the light path length was 1 cm. PerkinElmer Spectrum spectrometer recorded the Fourier transform infrared (FTIR) spectrum of synthesized GelMA and TPO nanoparticles frequencies ranging from 400 to 4000 cm^−1^. Hydrogen-Nuclear Magnetic Resonance (H-NMR) was performed using Bruker BioSpin GmbH instrument.

### Physio-mechanical properties of hydrogel

#### Swelling ratio, in vitro enzymatic degradation, mechanical compression test

Freeze-dried samples were weighed and immersed in water to study the effect of GelMA concentration on the swelling ratio of hydrogels. The samples were weighed after 24 h incubation at 37◦C. The swelling ratio was measured based on Eq. (1):

1 $$\mathrm{Swelling\,ratio }(\mathrm{\%}) =\frac{W2-W1}{W1 } \times 100$$

W1: freeze-dried sample, W2: after 24 h incubation.

Enzymatic degradation was chosen to evaluate the biodegradation rate and study the potential of hydrogel scaffolds for soft tissue regeneration. For this purpose, cylindrical samples were freeze-dried, weighted, immersed in water for 2 h at 50 °C until they reached the equilibrium swelling, and exposed to the 0.05% w/v collagenase in hanks buffer solution with 3 M CaCl_2_. Samples were monitored until they degraded thoroughly, and the weight loss was determined based on Eq. (2):

2 $$\mathrm{Weight loss }(\mathrm{\%}) =\frac{W1-W2}{W1 }\times 100$$

W1: equilibrium swelling weight, W2: monitored weight after exposure to the enzymatic solution.

A uniaxial compression test was also performed (Gotech AI3000/10 Kg Force) on cylindrical samples with 6 mm diameter and 3 mm thickness at a 10 mm/min strain (on five replicate samples in each group). Samples were prepared using a mold with the same size. Uncured hydrogels were poured into the mold, then subjected to the UV projector.

### Cell culture

Human breast adenocarcinoma cells, MCF-7, and Human umbilical vein endothelial cells (HUVECs-obtained from National Cell Bank of Iran) were harvested in high glucose DMEM and F 12 DMEM respectively and supplemented with 10% FBS, 100 IU ml^−1^ penicillin and 100 mg ml^−1^ streptomycin in T-25 cm^2^ flasks. They were maintained at 37 °C in a humidified atmosphere of 5% CO_2_, and the culture mediums were replaced every other day. At 90% confluency, the MCF-7 and HUVEC cells were detached by using trypsin into a single-cell suspension for 5 min, and to neutralize trypsin, 6 ml of growth FBS-containing mediums were added. For removing the trypsin and also dead cells, cell suspensions were centrifuged at 120 rpm for 5 min. Finally, supernatants were removed and cells were resuspended in 1 ml fresh culture mediums. Hemocytometer was used to count the cells.

### 3D cell spheroid culture

We fabricated 3D cell spheroids of the MCF-7 cell line by using our previously reported self-filling microwell device^24^. Briefly, agarose microwell was fabricated by casting the hot agarose solution on the 3D-printed mold. UV light sterilized agarose microwells for 45 min. Before cell seeding, the culture medium (DMEM high glucose) was added to the microwell to be completely swollen. MCF-7 cells (2 × 10^5^) were gently seeded in the loading site of microwell and incubated for four days without any movement. Three-D cell spheroids were ready to use after 1 week of incubation.

### Treatment of cells with TPO NPs and MTT assay

We set three time points (24, 72, and 96 h) and five concentrations (0.1, 1, 10, 100, and 1000 µg/ml) of TPO to study how NPs could interact with cells at different concentrations and times. In the 2D condition, 10,000, 7000, and 5000 HUVEC cells were seeded in the wells of 96 well plates and incubated overnight for attachment of cells. In the following, the culture medium was replaced with NPs diluted mediums and incubated to reach the time points. In the 3D culture condition, the culture medium of wells was replaced with NPs-diluted one and they were incubated until reached the planned time points. The effect of TPO nanoparticle treatments on the HUVECs and MCF-7 cell spheroid’s viability was measured by using the 3-(4,5-dimethylthiazol-2-yl)-2,5-diphenyltetrazolium bromide (MTT) assay to calculate cellular response at each desired treatment time and concentration. MTT stock solution was diluted in by the culture medium to reach the working concentration. Plates were incubated for 4 h and then the MTT solution was removed and 100 µl of DMSO was replaced. The plates were Shaked to dissolve the formazan completely in DMSO and then the optical density was measured by using a plate reader at 570 nm and changed into the cell viability using this equation:

$${\text{Viability }}\% \, = \,\left( {{\text{A}}_{{\text{treated cells}}} /{\text{A}}_{{\text{control cells}}} } \right)\, \times \,{1}00.$$

where A treated means absorbance of treated wells, and A control means absorbance of control. All of the experiment was done in triplicate.

### Live/dead staining

To examine the viability of the monolayer and tumor cells within the spheroid and also to determine the probability of cell death during the spheroid formation process in 4 days, the Live/Dead assay was conducted using 1 μM calcein AM and 4 μM ethidium homodimer-1 (Life Technologies kit) for 30 min at 37 °C. The whole spheroid staining and imaging process were performed on the microwell without the need for spheroid removal before staining.

### Flow cytometry

HUVECs were harvested in a six-well plate at the density of 200,000 cells per well. Cells were allowed to adhere overnight prior to exposure to nanoparticles. After incubation cells were exposed to TPO NPs at 100 µg/ml concentration, Irgacure 2959, and also culture medium without any particles for an additional 24 h. After exposure, the supernatant medium from each well was removed. Adherent cells from the same well were then trypsinized and transferred to the centrifuge. Cells and nanoparticles in each centrifuge tube were then pelleted and the supernatant was discarded. Pelleted cells were re-suspended in a solution containing a working concentration of Annexin and PI dyes and incubated for 0.5 h at room temperature prior to analyzing them by using flow cytometry.

### Cell adhesion on the 3D printed GelMA/PEGDA hydrogel by using TPO NPs

3D hydrogels were printed using a DLP 3D printing system using TPO NPs as a photoinitiator, GelMA-PEGDA as photocrosslinkable hydrogels, and food dye as a biocompatible photoabsorber (Food coloring has no significant effect on cell fate and they are safe to use in biomedical applications^25^. Also, they are water soluble and can be used with hydrogels^26^. After optimizing the conditions, 3D-printed hydrogels were free-dried and sterilized by utilizing UV light, and gently soaked in the culture medium. Then HUVECs (10 × 10^4^) were seeded on the surface of the scaffolds. After 24 h, the adhesion of cells was monitored by fluorescent DAPI staining, in which cells were fixed using glutaraldehyde and then dehydrated using ethanol, and finally, fixed cells were incubated with DAPI for 30 min at a dark room.

### Statistical analysis

Data were expressed as mean ± standard deviation (SD). Statistical significance among the experimental groups was determined using two-way ANOVA (* < 0.05: significant, * < 0.01: extremely significant).

## Results and discussion

### Characterization of TPO nanoparticles

Converting poorly water-soluble TPO into nanoparticles which can be easily dispersed in water by drying a microemulsion was reported previously^22^. Similarly, the nanoparticles were synthesized. The obtained powder from the lyophilization of an oil-in-water solution gives a clear aqueous solution (Fig. 1B) that can be used in many applications. In this synthesis, one of the most critical components is PVP, which acts as a crystallization inhibitor during dispersion in water. Investigating the morphology of synthesized nanoparticles has been evaluated using FE-SEM (Fig. 1C). The geometry of the nanoparticles confirms the microemulsion process. Structural analysis observed that the size of nanoparticles ranges from 50 to 150 nm, and the size distribution of nanoparticles was almost homogeneous. X-ray diffraction was used to study the resulting nanoparticles of TPO. TPO bulk exhibited a crystalline XRD pattern and sharp peaks in 2θ ~ 10°, 20°, and 18°, which were attributed to the crystalline structure of TPO bulk. The obtained TPO nanoparticles from described microemulsion are covered by PVP and SDS (Fig. 1D), which eliminates sharp crystalline peaks in the pattern and as a result of the amorphous structure, shows amorphous patterns (Fig. 1E). Several studies have shown that PVP undergoes intermolecular interactions with hydrophobic compounds and delays crystallization or increases the nucleation kinetic barrier^27–29^. Hydrophobic attractions are one of the interactions that occur between solvent and solute components. This interaction causes the formation of aggregated structures. Molecules of a solvent (especially water) have a strong bond. Only those solutes that can overcome the attractive force between water molecules are able to dissolve in the aqueous medium; otherwise, the water prevents them from spreading, and therefore aggregate structures are formed^30^. The micelle structure is one of these aggregate structures that is created when surface-active molecules are added to aqueous solvents. Surfactant molecules are amphiphilic, so they have a hydrophilic part and a hydrophobic part. When surfactant molecules are added to the aqueous medium, the hydrophilic ends are inclined towards the water, and water molecules strongly repel their hydrophobic ends. Therefore, aggregated structures are formed by adding appropriate amounts of surface-active molecules to aqueous solvents. Suppose these surface-active molecules are added to an organic solvent due to the high polarity of the solvent. In that case, the hydrophobic ends direct toward the solvent, and the hydrophilic ends gather together. Such a structure observed in organic solvents is called reverse micelle structure in which, unlike conventional micelles, surface-active molecules cover water spheres. This method is used to grow nanoparticles in an aqueous environment at room temperature^31^. As mentioned before and schemed in Fig. 1D, during the synthesizing process, TPO particles are covered by PVP, and a micelle structure results in nanoparticles of TPO. Figure 1F represents the FTIR spectrums of the TPO NPs structure. According to the results, N–C = O, C–C, C–N, C–H, C=O, and CH_2_ vibrations occurred in 572, 1017, 1286, 1373, 1646, and 2922 cm^−1^, respectively; which confirms the presence of PVP on the TPO NPs. In short, the synthesis of nanoparticles in the microemulsion method needs the combination of two microemulsions, one containing the first reactant and the other containing the second reactant. The reaction is carried out through the collision of two drops during the following steps:

- The movement of aqueous phase droplets in the emulsion and their collision
- The opening of the protective layer of surface-active material due to the collision and mixing of drops
- Penetration of reactive molecules from one drop to another
- Carrying out the reaction between the reactants, the formation of the nucleus and its growth
- Droplet separation

Surface adsorption of surface-active molecules on the nanoparticle surface prevents its excessive growth inside a microemulsion. Therefore, microemulsions can limit the size of particles to the desired value. Many factors, such as the ratio of the organic and aqueous phase, temperature, reactive compounds, and their amounts in the aqueous phase, affect the droplet size of the microemulsion and its phase behavior. Also, due to the existence of a wide range of surfactant molecules, the type and choice of surfactant affect the quality of the obtained particles, morphology, and size distribution. Due to the phase and thermodynamic behavior of the ternary system, the droplets prepared in the emulsion have a relatively uniform size distribution.

Finally, in the UV–VIS spectrums, we observed sharp peaks at 365 and 400 nm wavelengths (Fig. 1G). These peaks demonstrate the capability of TPO nanoparticles in light-based 3D printing or photopolymerization. The absorption of light by PIs affects the depth of light penetration in such a way that if the absorption is too high, the light cannot pass through the first layer of the light-curable resin, or if the absorption is low, the degree of conversion drops^32^.

### Cell toxicity of TPO nanoparticles

**Figure 2:**
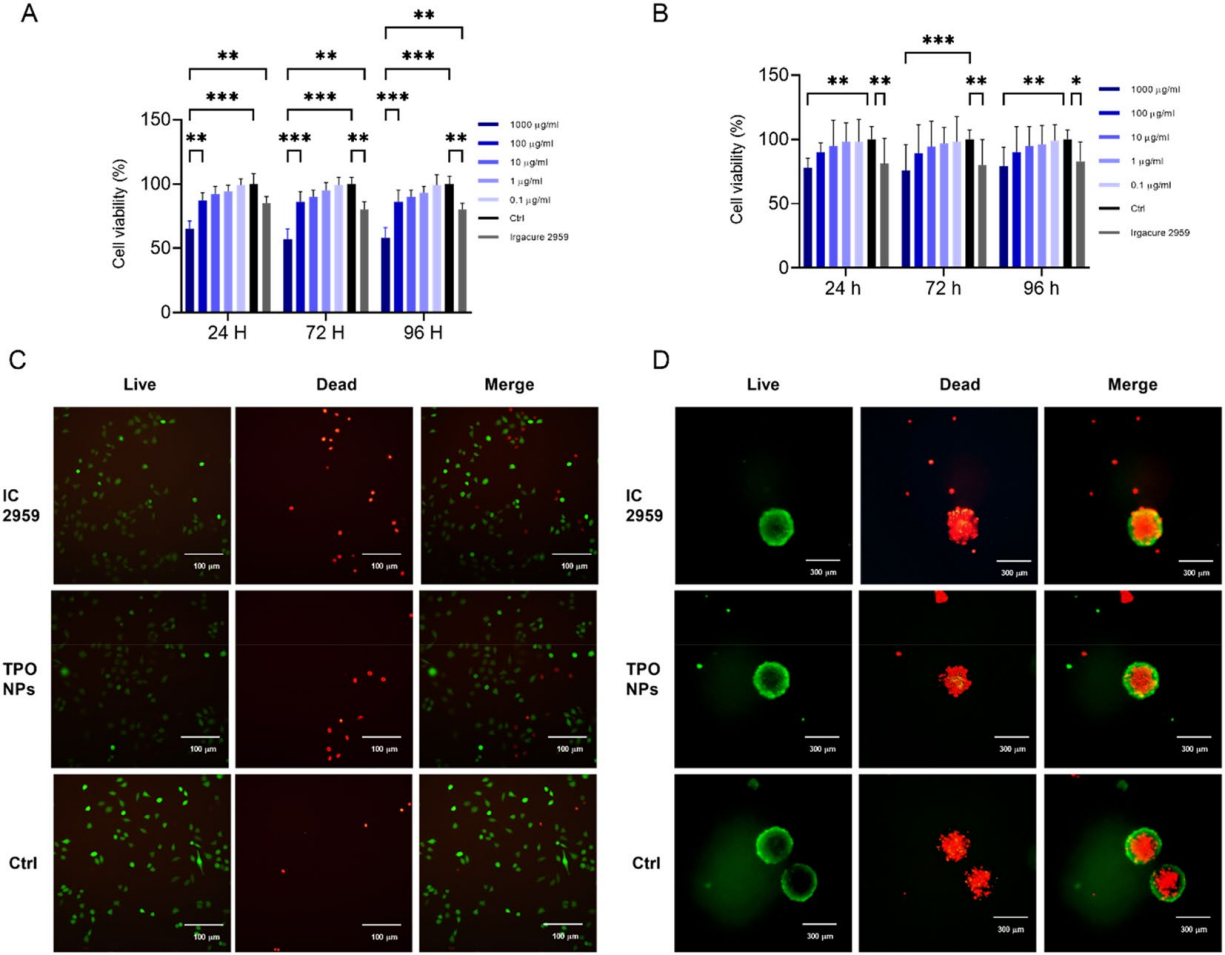
Cell toxicity assays of TPO NPs: (**A**) MTT assay on 2D cultured HUVECs, (**B**) MTT assay on 3D cultured MCF7s. All of treatments were done in triplicate and concentration of Irgacure 2959 was 10 µg/ml. (**C**) Live/dead staining on 2D cultured HUVECs, and (**D**) Live/dead staining on 3D cultured MCF7s treated with TPO NPs (10 µg/ml), Irgacure (10 µg/ml), and ctrl as control. Cells in 2D and 3D conditions were incubated for 24 h with treatments and then stained.

According to our knowledge, there is no study on the cytotoxicity of TPO NPs in a biomedical application where it has been used in the vast bioprinting fields as a photoinitiator. Cellular activities such as viability and proliferation are mainly affected by the nanoparticles when they are dispersed in a culture medium ^33^. In this study, the MTT assay was performed on HUVECs cells, which are known as healthy and non-cancerous cells, and also on MCF-7 cell spheroids as cancer cells. In this assay, the effect of different doses of TPO nanoparticles on HUVECs was evaluated at three different exposure times (Fig. 2A). According to the graph, with the decrease in the nanoparticle concentration, the viability rate at a specific exposure time has increased significantly, and as it is shown, in 1 μg/ml and lower concentrations, they did not cause toxicity to HUVECs compared to the control sample. At 1000 μg/ml concentration, it can be seen that the cell viability decreased after 4 days. The results of MTT show that this nanoparticle has no significant toxicity compared to the control sample at concentrations below 100 μg/ml and can be safe for bioprinting applications and also for scaffold fabrication. The results of this study suggest that if the nanoparticle remains unreacted in the environment during the working process of the photoinitiator, it cannot induce toxicity or damage to the cultured cells on the medium. The toxicity of photoinitiators is one of the primary challenges in tissue engineering. For example, the common photoinitiator Irgacure 2959, which has many uses as a crosslinker in photosensitive hydrogels, due to its low solubility in aqueous environments and biological buffers and high toxicity, may not be successfully used in tissue engineering applications^34,35^. The supply of PI with high solubility in water, along with strong performance and biocompatibility, has been one of the constant challenges of tissue engineering in the field of photo-crosslinkable hydrogels. So far, a PI with the mentioned feature has been introduced. Due to its high solubility in water and biological buffers, a stronger performance than the commonly used PI, Irgacure 2959, and very low toxicity in the concentrations used in the crosslinking process, TPO nanoparticles can be introduced as an effective initiator as a binder. The viability of HUVECs after treatment was also imaged by live–dead staining. As depicted in obtained live dead pictures, TPO NPs are not toxic compared to commercially available PI, IC 2959, and control samples, and the results are in line with the MTT assay.

Two-dimensional cell culture is an in vitro model to research cell toxicity of implants or drugs. Unfortunately, animal studies or clinical trials do not confirm the results of two-dimensional monolayer cultures as they are able to simulate extracellular microenvironments and mimic cell–cell interactions. Therefore, the development of cell-cultured models is necessary to bridge the gap between monolayer cell studies and animal experiments^36^. 3D tumor models provide a valuable tool for in vitro studies. Compared to conventional monolayers, these models can mimic cell microenvironments. Cell–cell and cell-extracellular matrix interactions promote the function and simulation of the natural structure of the original tissue. The effect of this phenomenon is not correctly reflected in monolayer cell culture. In 3D bioprinting, cells interact with other cells and also the matrix. The endpoint of TPO NPs would be in the bioprinting application. To further study the toxicity of TPO nanoparticles, 3D MCF-7 spheroid models have been used. As Fig. 2B represents, cell viability was reduced only in the sample with 1000 μg/ml NPs in 24 h. In lower concentrations, not only did they not cause toxicity, but they did not inhibit spheroid growth, and this trend can be seen in the third.

Live–dead staining of cells in 2D and 3D conditions was efficient in visualizing the effect of TPO NPs on cellular mortality. As can be seen in Fig. 2C, the population of dead HUVECs treated by TPO NPs is lower than Irgacure 2959 in constant concentration and incubation time (10 µg/ml and 24 h). However, a large number of MCF-7 cells have already died because of hypoxia mimicking the natural tumor structure, the addition of TPO NPs did not induce more toxicity in comparison with Irgacure 2959 (Fig. 2D). The result of live–dead staining supports MTT findings in which there was negligible toxicity induced by TPO NPs.

**Figure 3:**
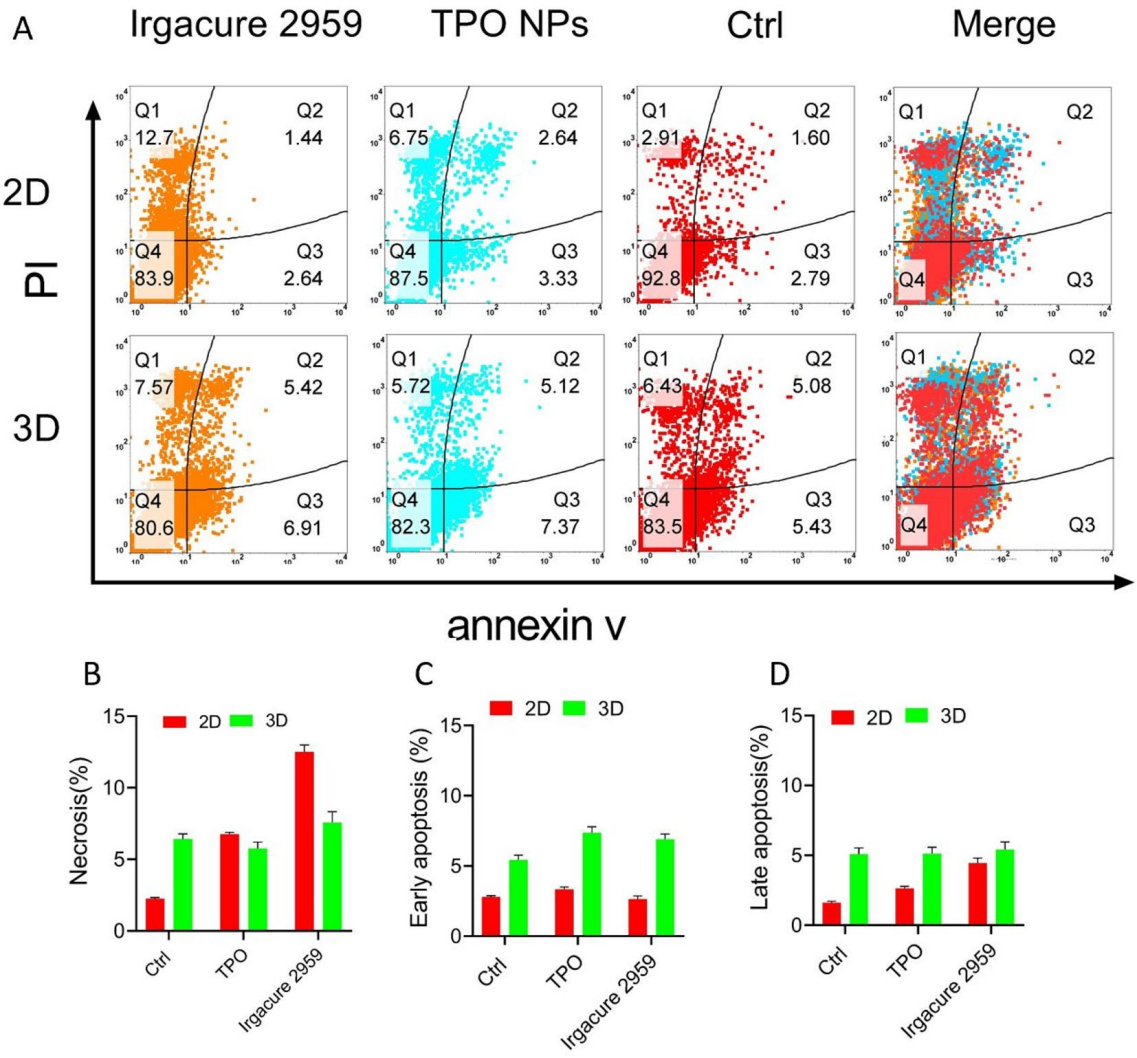
Flow cytometry analysis of the HUVECs apoptosis and necrosis after TPO NPs or IC2959 treatments, with annexin and PI staining. The concentrations of TPO NPs and Irgacure 2959 were constant at 10 µg/ml.

While photoinitiators seem to be cell-friendly, understanding the mechanism of death in cells that are treated by photoinitiators is critical. Cell death (apoptosis and necrosis) occures when they are treated with NPs. Flow cytometry is a tool to assess cell toxicity induced by nano and microparticles^37–39^. We used flow cytometry to study how TPO NPs induce cell death. In Fig. 3A, each plot is divided into four quadrants; Q1, Q2, Q3, and Q4 represent necrosis, late apoptosis (PI), and early apoptosis (annexin). An individual cell will be counted in one of the four quadrants based on its dye uptake. Based on the results, simplified in bar charts shown in Fig. 3B–D, the apoptosis and necrosis induced by TPO NPs is less than IC 2959, and in some cases, it is as same as control samples.

One of the main issues in the bioprinting field is the toxicity caused by photoinitiators, which threatens cell viability^15,40,41^. The results of two-dimensional and three-dimensional studies reveal that this nanoparticle can be used not only for light-based crosslinking of hydrogels but also for bioprinting in the presence of cells and biomolecules. However, other studies, including gene expression and protein synthesis, are needed to complete these studies to prove that this nanoparticle does not affect cell metabolism.

### TPO NPs and their application in 3D printing and photopolymerization

**Figure 4:**
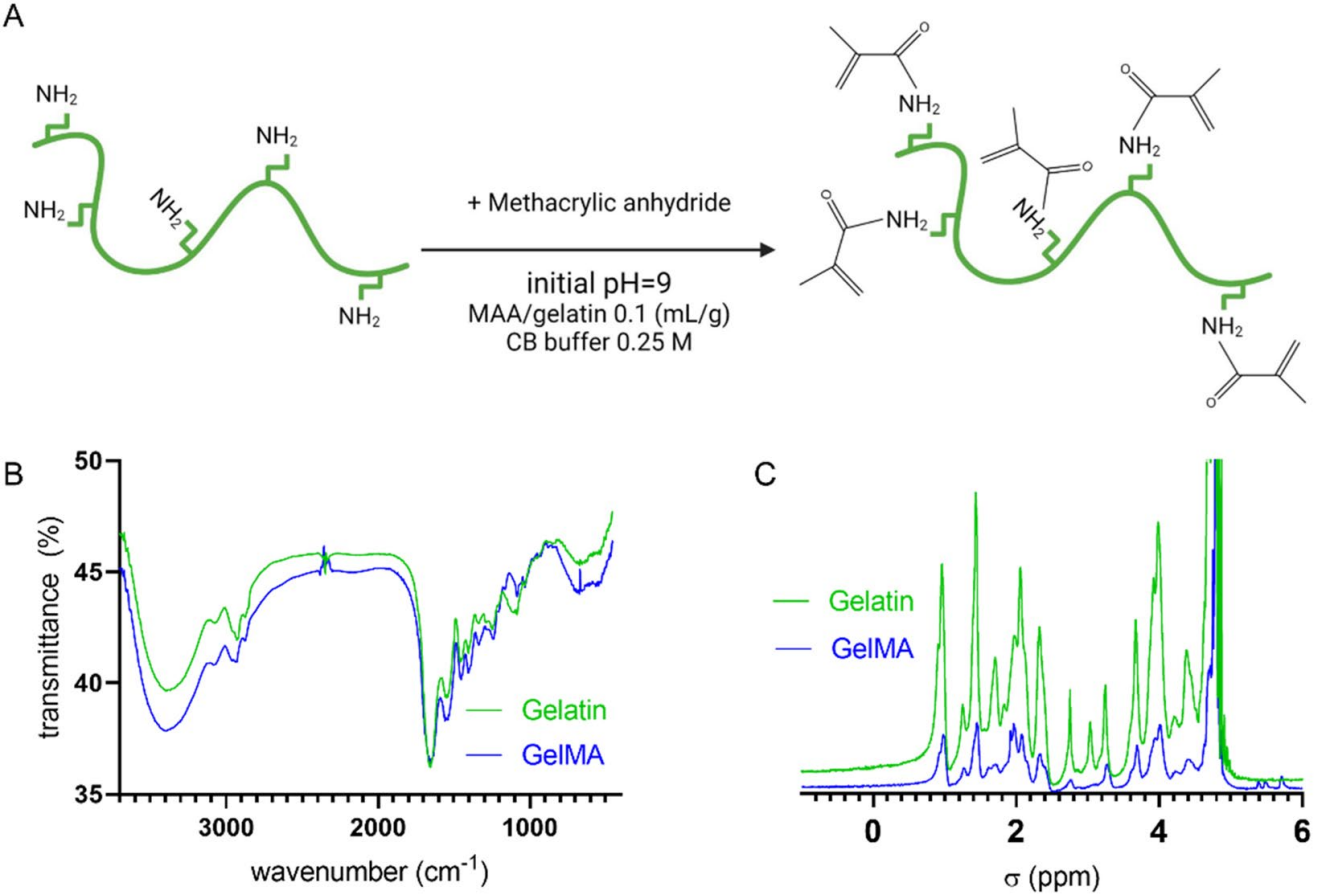
GelMA synthesis characterization: (**A**) a schematic of gelatin functionalized with NH2 groups, (**B**) FTIR, and (**C**) H-NMR spectrum of gelatin and GelMA.

For the proof of principle, we investigated the applicability of synthesized TPO NPs in photocrosslinking. However pristine TPO is a commercial photoinitiator widely used in the industry, and its low water solubility is a concern. To investigate the functionality of TPO NPs, they were used in hydrogel 3D printing. For this purpose, we synthesized gelatin Methacrylate (GelMA, Fig. 4A), a widely utilized hydrogel in tissue engineering^23,42–46^.

Figure 4B, C shows the results of GelMA characterization. Both FT-IR and H-NMR confirm the structure of obtained GelMA. Also, DS of synthesized GelMA was calculated using the changes in the area of 2.8σ, which was 96% for this synthesized GelMA^23,44,47^.

Using TPO NPs as a PI in light-based 3D printing is proof of our study. As discussed in the first section, to print a hydrogel structure using a DLP printer, the resin must contain three important parts: polymer, photoinitiator, and photo absorber. In this project, the design of the resin was examined step by step, and at the end, after the synthesis and examination of the required materials, the resin was optimized for a 3D DLP printer.

**Figure 5:**
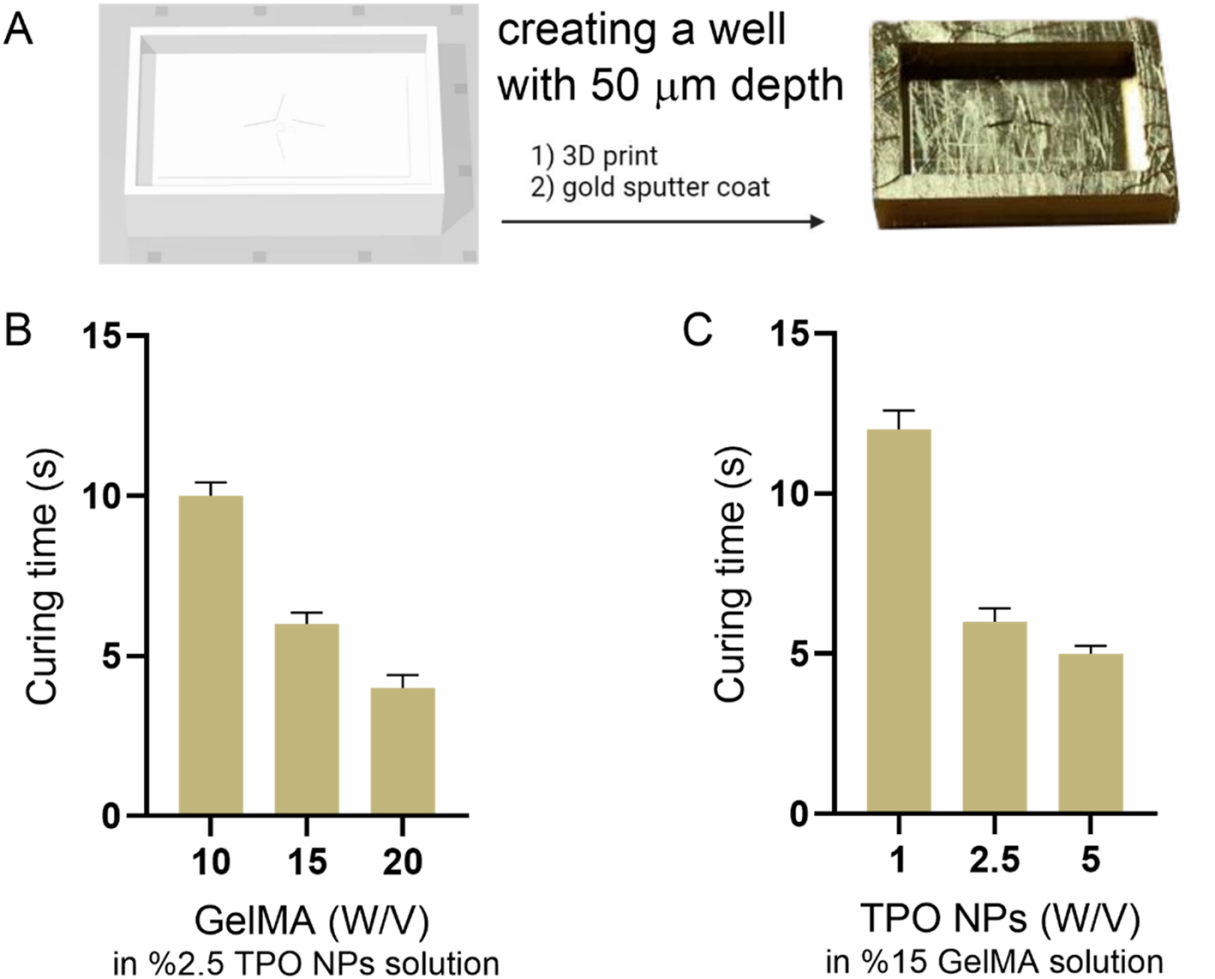
Curing time optimization of bioresin: (**A**) CAD and printed well for optimization, (**B**) effect of GelMA concentration on curing time, and (**C**) effect of TPO NPs concentration on curing time.

The first step in the printing process is optimizing curing time. This time should be as short as possible to optimize the physio-mechanical properties of hydrogel and the high printing speed. Molds with pre-defined wells or micrometric patterns can be used to optimize curing time^48^. In this project, the optimum curing time was obtained by a mold printed using commercially available DLP resins (Fig. 5A). The well with a thickness of 50 µm was ready to be used. Different batches of bioresin with different concentrations of GelMA as well as PEGDA were prepared and poured into this well, and the gelation time was evaluated in the presence of the UV light projector. The results showed that by increasing the concentration of GelMA, the curing time is reduced (Fig. 5B). In this study, the concentration of TPO NPs was %2.5 w/v. Also, food dye was used as a photo absorber. The effect of TPO NPs concentration on curing time was examined in %15 w/v GelMA solution (Fig. 5C). As the difference between the curing time of solution with %5 and %2.5 of TPO NPs was insignificant, it is not necessary to use a higher concentration of NPs to prevent toxicity. So, by choosing the optimal composition (%15 w/v GelMA and %2.5 w/v TPO NPs), the curing time for the 50 µm layer reached 6 s.

### Physio-mechanical properties of hydrogel

One of the important properties of hydrogels is their mechanical properties which can be measured by different tension and compression tests. In tissue engineering, mechanical properties should be comparable to tissue to ensure a more uniform stress distribution at the implant and minimize the stress at the tissue interface^49,50^.

**Figure 6:**
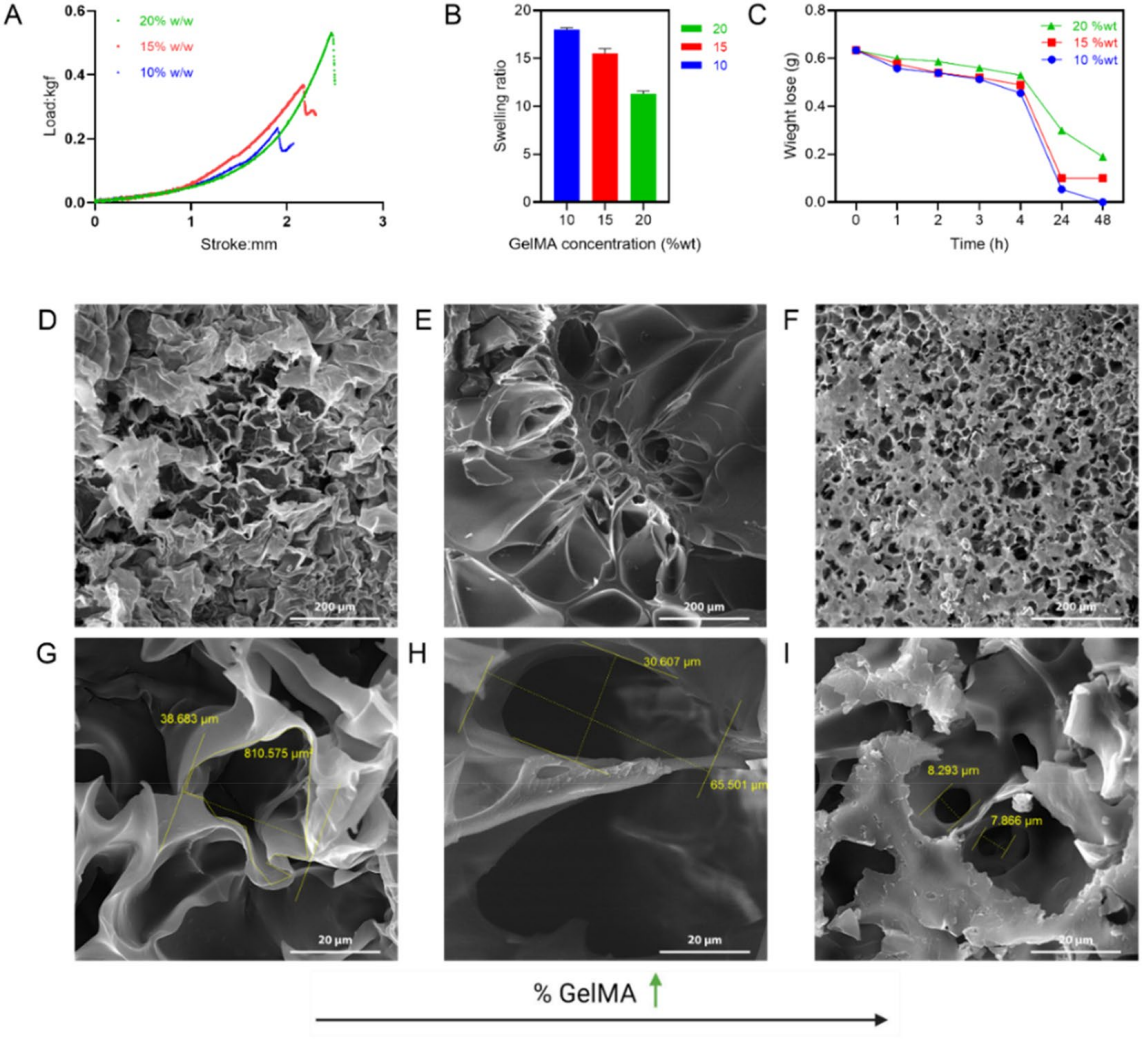
Effect of GelMA concentration on (**A**) mechanical compression test, (**B**) swelling ratio, (**C**) biodegradation rate, and porosity of cured hydrogels (**D,G**) 10%, (**E,H**) 15%, (**F,I**) 20% w/v GelMA.

In this research, the compressive modulus was measured at different concentrations of GelMA. As shown in Fig. 6A, the yield stress increased significantly with the increase in GelMA concentration, so that it is about 2.2 newtons for %10 w/v GelMA while it is about 5 N for %20 w/v GelMA. The compression test was also performed on GelMA hydrogel swelled in water. The results indicated that with the increase in the amount of polymer, more chains could withstand the applied pressure; as a result, the yield stress increases, and the hydrogel becomes stiffer. The following shows that this increase in the concentration of GelMA has caused the pores of GelMA hydrogel to become smaller. Therefore, it can be said that the reduction of these pores can improve the mechanical properties of the hydrogel.

The swelling ratio of these hybrid hydrogels was also investigated after swelling overnight. Studying the swelling ratio of hydrogels is necessary since it affects other properties. The swelling ratio in hydrogels is affected by the pore size and the interaction between the solvent and the polymer^51^. The swelling of 10, 15, and %20 w/v GelMA samples was investigated (Fig. 6B). The concentration of PI and the exposure time were also considered constant. It was observed that with the increase of the concentration of GelMA, the swelling ratio decreases dramatically; it decreases from 18.2 ± 3.7 for %10 to 5.3 ± 1.7 for %20 w/v GelMA. It has been reported that in GelMA hydrogel, the substitution percentage significantly affects its swelling properties^52^. In this work, many parameters, such as the amount of added methacrylic anhydride, the DS, the concentration of PI, the curing time, and the exposure time, can affect the mechanical properties. It can be seen that the increasing concentration of GelMA has increased the crosslinking density in the sample, which will also be discussed in the next part. It can be seen that it improves mechanical properties. Generally, the swelling ratio and mechanical properties in hydrogels have an inverse relationship. Also, as previously shown, the decrease in the pore size makes it harder for liquids to enter the hydrogel network, so the amount of absorption and consequently, the swelling ratio will decrease.

Biodegradability is another critical factor in tissue engineering that should be considered in the design and application of scaffolds. In this part, we investigated the enzymatic degradation of hydrogels in the presence of collagenase. Collagenase is an enzyme that dissolves collagen and is widely used in different studies^49^. The results showed that with the increase in the GelMA concentration, the hydrogel degrades more slowly than the sample with a lower concentration (Fig. 6C). The biodegradability rate depends on the concentration of the primary polymer and the crosslinking density. With a slow biodegradation rate, GelMA has great potential to be used in topical drug delivery, where sustained release is required. This hydrogel can release the drug in a controlled and long-term manner. In tissue engineering, after cell adhesion, growth, proliferation, and differentiation, the development of extracellular matrix becomes a challenge, which can be manipulated by altering GelMA-based hydrogel degradation^53^.

SEM spectroscopy was performed on hydrogel samples with different GelMA concentrations to further study the structure (Fig. 6D–I). As can be seen in the pictures, reducing GelMA concentration in the composition has significantly affected pore size in the structure. In Fig. 6I, the average diameter of pore size is 8 µm for the samples with 20% of GelMA, whereas they are 40 µm in diameter when the GelMA concentration decreases to 5% (Fig. 6G).

**Figure 7:**
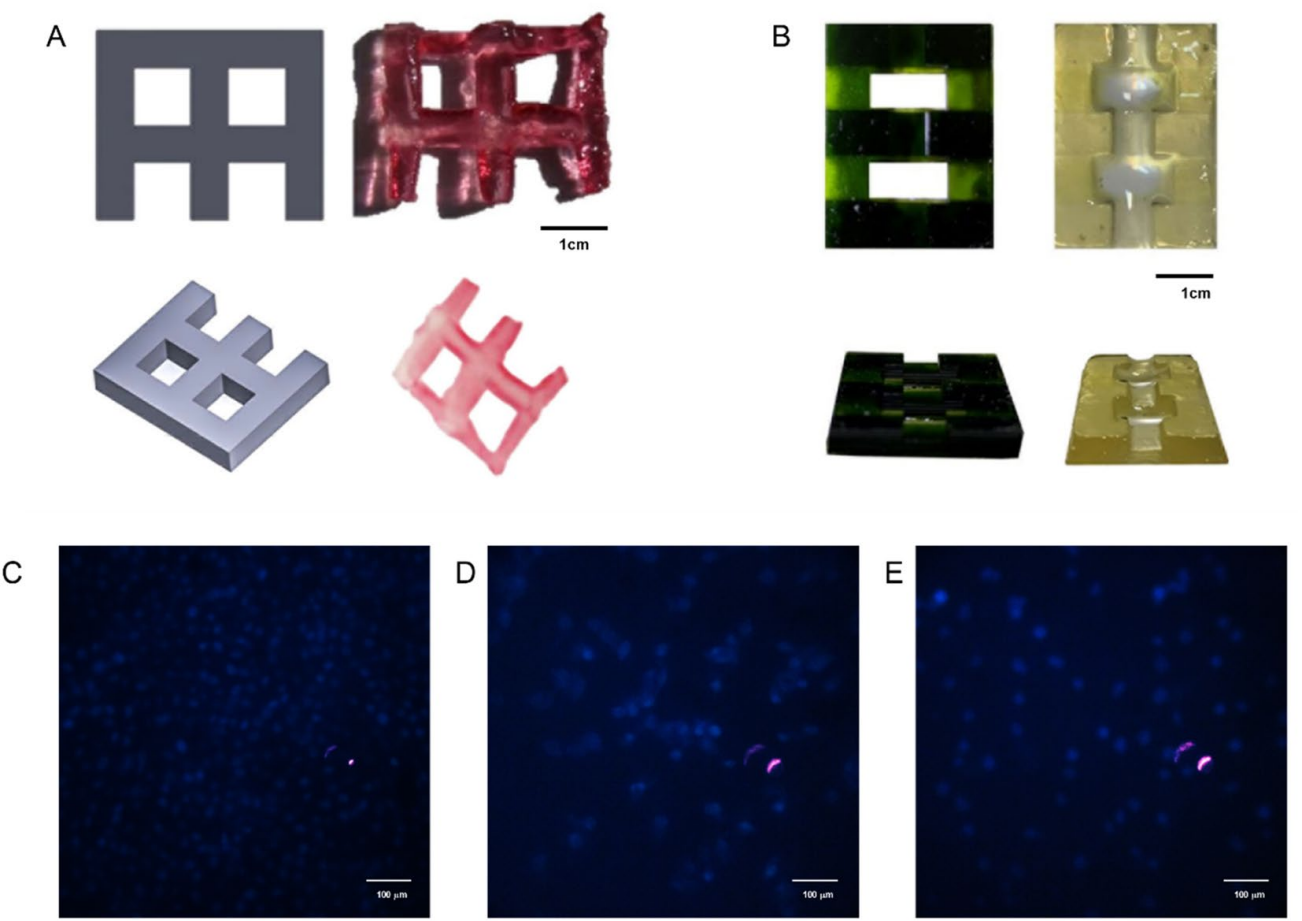
(**A**) CAD (left) vs. printed hydrogel (right), DAPI staining of cultured HUVECs on printed hydrogel containing, (**B**) printed object: commercial resin (left) vs. bioresin (right), (**C**) 10%, (**D**) 15%, and (**E**) 20% w/v GelMA.

### Printability and cell adhesion

3D printing of bioresin with 15% GelMA was performed with high accuracy compared to the printing of the commercial resin of the device (Fig. 7B) and the CAD design (Fig. 7A). Not only will these geometries allow us to see the ability of 3D printers to define angles, but also they enable us to investigate under-or-overcuring in the structure. the printing depth was adjusted to 50 μm. The samples were printed with a three-layer base at 1000 ms exposure time per layer, while the main body was printed using an 600 ms exposure time. Following the DLP 3D printing process, the printed scaffolds were immersed in phosphate buffered saline (PBS) at a temperature of 40 °C for 5 min to eliminate any uncrosslinked inks (DLP apparatus was purchased from Kavoshlaser). To further study, HUVECs were cultured on the printed scaffolds with different GelMA concentrations (10, 15, and 20% shown in Fig. 7C–E, respectively), and stained with DAPI. Images of stained nuclei show the distribution and attachment of cells to printed hydrogels after 24 h. This means that the printed hydrogel of optimized bioresin using synthesized GelMA and TPO NPs are non-toxic and can be used as scaffolds in tissue engineering.

## Conclusion

In summary, to address the issues in light-based bioprinting and tissue engineering applications of light-based 3D printing, TPO was converted into water-soluble, efficient, and non-toxic nanoparticles to be used as PI. We characterized, evaluated the cell toxicity, and used these nanoparticles in light-based 3D printing with GelMA hydrogels (via a DLP printer). The synthesized TPO NPs can be used in bioprinting and tissue engineering applications, as shown by the results of this study.

## Data Availability

The datasets generated during and/or analyzed during the current study are available from the corresponding author on reasonable request.
